# Quality of Service (QoS) improving schemes in optical networks

**DOI:** 10.1016/j.heliyon.2020.e03772

**Published:** 2020-04-16

**Authors:** Dawit Hadush Hailu, Gebrehiwet Gebrekrstos Lema, Berihu G. Gebrehaweria, Samrawit H. Kebede

**Affiliations:** School of Electrical and Computer Engineering, Ethiopian Institute of Technology–Mekelle (EiT-M), Mekelle University, Ethiopia

**Keywords:** Electrical engineering, Computer science, Network (computer science), Computer engineering, Communication system, CPT, OPS, Optical networks, QoS

## Abstract

In optical networks, such as OPS/OBS, the network results into significant loss in the network layer. When the loss significantly deteriorates the QoS by increasing the Bit Error Rate (BER), a viable approach can be used to increase the performance. This paper presents state of the art of Quality of Service (QoS) schemes used for improving the performance of optical networks. Furthermore, some possible applications and performance data are summarized based on Packet Loss Rate (PLR), secrecy, survivability and other parameters. The different states of art methods proposed by several authors are compared with Coded Packet Transport (CPT) scheme. We believe that this study is valuable to researchers envisaging a novel approach to enhance the performance of optical networks for telecommunications networks of the future.

## 1 Introduction

**Figure 1:**
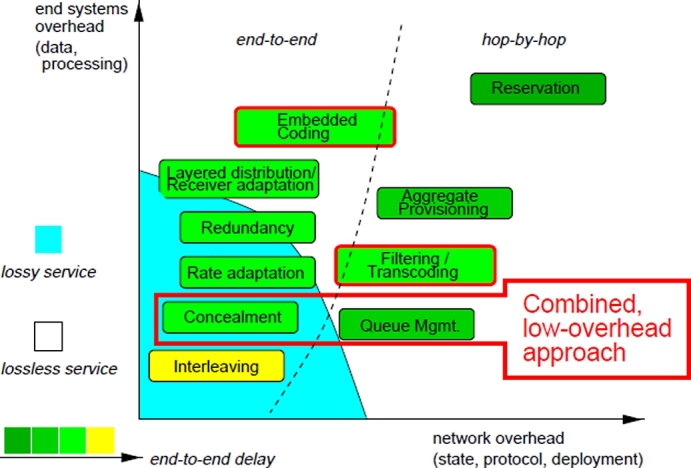
Taxonomy of loss treatment schemes.

Recently, optical networks based on Dense Wavelength Division Multiplexing (DWDM) techniques are already in use in the current commercial telecommunication networks as a primary transmission medium. These techniques employ light wavelengths to transmit data from multiple wavelength channels or different sources over a single strand of optical fiber. In traditional networks, information has been transported from source to destination using circuit switched fiber optic networks. Though guaranteed QoS, low delay variation, low delay, and synchronization support are the essential and vital features of Optical Circuit Switched (OCS) networks, they are not bandwidth efficient for bursty IP traffic [1]. As a result, the rapid growth of IP traffic volume with the resulting demand for capacity leads to the migration of the networks from optical circuit switched networks to packet based optical networks. This inevitable shift brings better bandwidth utilization and lowers cost by introducing statistical multiplexing; however, the low delay, low latency variation, and guaranteed QoS are only offered by OCS technologies and are still essential for mobile fronthaul [2], [3], backhaul, metro, and transport networks. Both Optical Packet Switched (OPS) [4], [5] and Optical Burst Switched (OBS) [6] networks introduce Statistical Multiplexing (SM) to overcome the inefficient utilization of bandwidth. Packet losses caused by contention when two or more packets are arrived for the same output wavelength at the same time, are a crucial issue in such networks. In order to combat this issue, various contention resolution mechanisms/approaches such as buffering with optical buffering, Fibres Delay Lines (FDL), burst segmentation [7], deflection routing [8] and wavelength conversion are required. With regard to the deployment of Internet real time services, packet loss has been considered as a major problem. Fig. 1 presents a generic approach to packet loss avoidance and recovery techniques in the internet [9], [10]. Those techniques may operate either on hop by hop or on an End to End (E2E) basis. In end to end approach, the concern is implementing robust end system protocols and mechanisms, not in the network while the hop by hop approach involves active participation of the network at different levels to achieve better end to end delay and lossless service. The overall node deployment and scalability of the network are highly influenced by the associated overhead of both approaches. Considering scenarios with low bandwidth and numerous flows, scalability is a major concern as the approaches high per flow state overhead (reservation), redundancy mechanism, interleaving and receiver-based concealment.

In order to support and satisfy the above requirements in multi-service network simultaneously, improvements have been made to the optical packet switched networks to accommodate high throughput and effective QoS capability [5], [11]. The improvements have introduced hardware and software complexity which in turn result in expensive operational and capital cost of the techniques. Thus, a new concept of hybrid switch networks integrating both OCS and OPS technologies is introduced. It is proposed to provide support for various service requirements and efficiently utilize the bulk capacity of optical networks [12].

Moving from this background, the main objective of this paper is to study various redundancy schemes being used in telecommunication systems that can be used to improve QoS. In the first section, the concept of QoS is introduced and the comparison of several switching technologies are summarized in Section 2. After presenting the state of the art of QoS improving contention schemes in section 3, we compared the techniques with CPT scheme. Finally, section 5 concludes the study.

## 2 A comparison of switching technologies for WDM

Comparing the three approaches of switching technologies, Table 1 shows the granularity, utilization and complexity, bandwidth, setup latency, switching speed, processing overhead and traffic adaptability are summarized aspects of the different switching technologies. Looking into the table, an OBS technology is a compromise between a OCS and OPS i.e. it constitutes important features of both OCS and OPS.

**Table 1:** A detailed comparison of switching technologies for WDM.

Properties	Switching technology		
	OCS	OPS	OBS
Utilization	Poor	High	Moderate
Bandwidth	Low	High	High
Setup latency	High	Low	Low
Granularity	Coarse	Fine	Moderate
Switching speed	Slow	Fast	Medium
Processing overhead	Low	High	Low
Traffic adaptability	moderate	High	High
Complexity	Low	High, not mature	Moderate

Considering the above points in Table 1, it should be evident that an OBS network is a promising technology for the future internet network with a medium switching speed. It should also be evident that an OPS network is a promising technology, but it demands high overhead processing.

**Figure 2:**
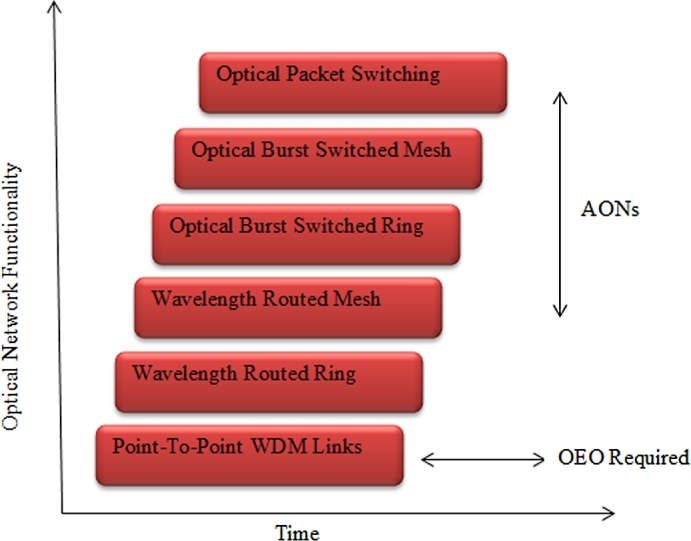
Evolution of optical networks.

The evolution of an optical network starting from point to point WDM links to optical packet switching is shown in Fig. 2. It covers from today's Point to Point (P2P) WDM links over add/drop multiplexers and cross-connects (CC) for ring and mesh networks to optical networks with higher reconfiguration speeds such as optical packet switching (OPS). As it can be seen from the evolution, OPS seems to be a hot research topic in the future and promising technology for optical networks. All optical networks include both Wavelength Routed Optical Networks (WRON) and Optical Packet/Burst Switched networks (OPS/OBS). In general, the figure depicts that the optical packet functionality has increased from P2P WDM link to optical switching.

## 3 QoS improving schemes: state-of-the-art

In the following sub-sections, the CPT is compared to some schemes.

### 3.1 Error concealment

The main challenges of most of the communication systems are bit insertion, bit deletion and bit inversion. Though the FEC and ARQ are broadly used for error control purposes, thy are not well exploited for video transmissions. Due to the low latency QoS requirement, the video transmission cannot accommodate an increased number of ARQs to guarantee the reliability of the communication. Hence, for video signal transmission, different error reduction techniques are developed in the state of the arts. In other words, when a packet is transmitted over a long distance, the resulting packet may be lost or erroneous. However, due to high delay and long distance transmission, the lost packet cannot be re-transmitted. Alternatively, the error concealment approach is proposed [13] which uses the preceding and/or the succeeding packets of the actual data to control the error issues [14]. The three error concealment techniques [13] are forward error concealment (FEC), post-processing error concealment and inter-active error concealment. The three error concealment techniques are also accomplished at the encoder, decoder and jointly encoder and decoder, respectively. For easier error retrieval purpose, the FEC technique introduces redundant data at the encoder. There are also variety of redundant introduction mechanisms including transport prioritization coding [15], joint source and channel coding, multiple description coding [16] and robust entropy coding. Besides, in the interactive error concealment, the interaction between coder and decoder enhances the error suppression competence of the system. They normally use channel feedback to combat the error issues. The error concealment techniques categorized under the interactive error concealment are adaptive transport, selective encoding [17], retransmission without waiting [18] and prioritized multicopy retransmissions. On the other hand, for the best possible error free reception, the postprocessing error suppression is used at the decoder. The spatial and frequency-domain interpolation, motion compensated temporal prediction [19], projection onto convex sets, maximally smooth recovery [20], recovery of motion vectors and coding modes are examples of the postprocessing error suppression technique.

### 3.2 Shared Packet Loss Recovery Scheme (SPLR)

**Figure 3:**
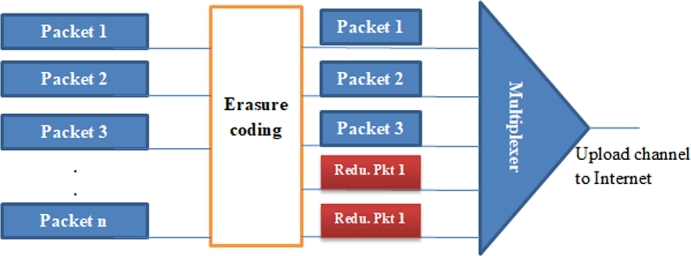
Shared packet loss recovery scheme.

The erasure code technique is also used based on Shared Packet Loss Recovery Scheme (SPLR), [14], to add redundant packets in the ingress node, and the outgoing streams share these packets to recover the lost packets. For *n* packet sets having pt packetization period, an erasure code is applied to all the packet sets coming out of the ingress node, shown in Fig. 3. The SPLR outperforms many packet loss recovery techniques including error concealment. It also introduces relatively limited recovery delay. On the other hand, the SPLR is effective in the one point sending multiple streams. When the ingress node *n* becomes larger, the performance difference between the scheme and the existing one becomes smaller. Hence, the usage of redundant packets in different methods is small. In practice, *n* would not be large because admission control would limit the number of telephone sessions for good quality of service. To illustrate it numerically, suppose the ingress node has a bandwidth of n=20 packets per $p_{t}$. We use the term bandwidth as the maximum number of packets that come out of ingress node in a pt so that every outgoing packets needs a bandwidth of 1. The ingress node employs an erasure coding on data packets k=15 packets, and adds redundant packets r=5. Thus, the ingress node forwards these 20 packets over a network to the destination node. The egress node now has to recover any of 15 packets out of 20 packets for recovering all the lost packets [21].

The scheme introduced a low probability of packet loss because several packets reaching at an ingress node can share the redundant packets for recovery of the packets, and involves a small recovery latency.

### 3.3 Hybrid Packet Loss Recovery (HPLR)

To minimize the packet loss possibility [21] and to enhance the throughput the hybrid packet loss recovery is used. The HPLR applies a silent features of error concealment and packet loss recovery technique. This is used to recover errors in the VoIP packets lost IP networks. In case of the HPLR, the neighboring VoIP packets of preceding and/or succeeding packets of the same data set can be correlated to each other. This implies that the erasure coding might not be required for the recovery of VoIP packet drop in IP network. Instead, error concealment is used to recover these packets, thereby reduction of sending unnecessary redundant packets. If the VoIP packets of preceding or succeeding packets of the same data set have no sufficient correlation, an SPLR is applied [21].

### 3.4 Network Layer Packet Redundancy Scheme (NLPRS)

In the OPS/OBS network, the packet loss due to contention, at the network layer, is the major QoS problem. In this network, when two or more packets tends to leave the node on the same output interface, on the same wavelength at the same time, contention occurs [22]. It causes a number of packets to drop in the network which increases the packet loss ratio (PLR). To avoid this situation the author [23] investigated the Network Layer Packet Redundancy Scheme (NLPRS). With the NLPRS, the redundant packet packets are extracted from the data portion of the incoming nodes. The size of the redundant packets must be as large as the biggest possible packet [23]. Then, the redundant packets are directed to the outgoing nodes. Thus, due to contention, some packets can be dropped at the outgoing node as the lossy network increases the packet drop rate.

In NLPRS, every activity is done in the ingress and egress node of the OPS core networks. It employs a Reed Solomon coding. The ingress node is responsible for constructing $r_{s}$ redundancy packets out of $m_{s}$ data packets received from the network to form a packet set having $r_{s}+m_{s}$ packets. When all packets are received, creation of the $r_{s}$ packets is performed by copying the data packets before they are scheduled. After creating $r_{s}$, its size should be equals to the longest packet in the packet set. Here, packets having common destination node are categorized into the same packet set. After the $r_{s}$ is created, it is made ready for sending using the outgoing node where the potential reconstruction is performed if any packet loss has occurred.

Due to contention, some packets can be dropped at the outgoing node as the lossy network increases the packet drop rate. Received packets at the egress node are denoted $r_{r}$ ($r_{r}\leq r_{s}$) and $m_{r}$ ($m_{r}\leq m_{s}$). The NLPRS packet recovery Scheme described in Algorithm 1.

**Algorithm 1:**
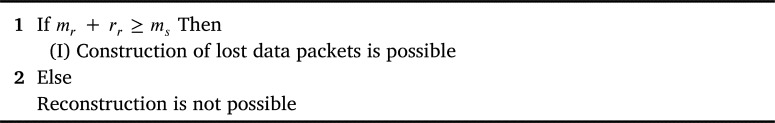
NLPRS lost packet recovery.

**Figure 4:**
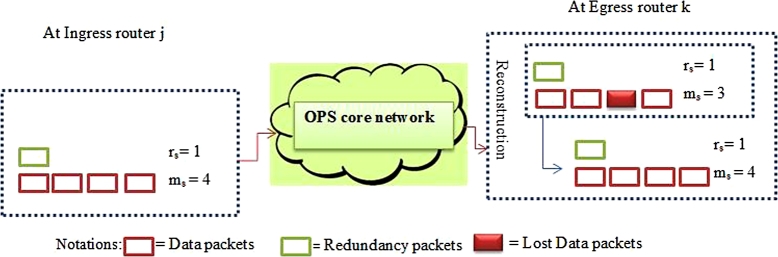
Network Layer Packet Redundancy Scheme (NLPRS).

Fig. 4 presents an example where $m_{s}=4$, $r_{s}=1$, $m_{r}=3$ and $r_{r}=1$. The example illustrates possible reconstruction of lost data packets as the $m_{r}+r_{r}=3+1=4\geq m_{s}=4$, which ultimately results in no data packet loss when the NLPRS is utilized. In order to reduce data packet loss, NLPRS use the redundancy effect for recovering the lost packets. When the offered load in the optical network is increased, the efficiency of NLPRS is reduced as it increases burstiness. However, if used properly, the NLPRS reduces the packet loss rate significantly with several orders of magnitude. Its performance depends on network size, *m* and *r*, the system load, data packet arrival process, redundancy packet scheduling mechanism and packet length distribution.

The working principle of NLPRS scheme for improving the overall performance of optical networks is described as follows.

Firstly, redundancy packets (*r*) are added to a set of *m* data packets. It assumed that $m_{s}$ are offered to an E2E path $\pi_{k}$ according to a Poisson arrival process with constant arrival intensity equal to $\lambda_{k}$. In OBS network, there no queuing; the arrival process is the sum of a individual Poisson in output link $e_{i}$ and its blocking probability $B_{i}$ is calculated according to the Erlang loss formula given in Equation (1) [23]:

(1) $$B_{i}=E_{N_{i},A_{i}}=\frac{\left({\left(N_{i}A_{i}\right)}^{N_{i}}/N_{i}!\right)}{{\sum_{j=0}^{(N_{i}}N_{i}A_{i})}^{j}/j!}$$

The normalized system load on $e_{i}$ is equal the arrival intensity for all paths flowing through $e_{i}$ after subtracting the amount of traffic lost due to contentions in output links traversed before arriving to $e_{i}$. This reduced load on $e_{i}$ is calculated as in Equation (2)

(2) $$A_{i}=\frac{1}{\mu N_{i}}(\sum_{\pi_{k}\in R,e_{i}\in e^{k}}\lambda_{k}\prod_{p=1}^{C}(1-I(e_{p},e_{i},\pi_{k})E_{N_{p},A_{p}}))$$

In order to calculate the E2E PLR for traffic flowing on path $\pi_{k}$, we must take into account the PLR on every output link traversed by the E2E path $\pi_{k}$, i.e.

(3) $$B(\pi_{k})=1-\prod_{e^{k}}(1-B_{i})$$

where $B(\pi_{k})$ is the E2E PLR for traffic flowing on path $\pi_{k}$.

When *r* redundancy packets are added to m data packets, the arrival rate changes from $\lambda_{k}$ to $(\lambda_{k}(r+m))/m$. In order to reflect the additional load imposed by the Redundancy Packets. Hence, the new normalized system load on $e_{i}$ is given as:

(4) $$A_{i,r,m}=\frac{1}{\mu N_{i}}(\sum_{\pi_{k}\in R,e_{i}\in e^{k}}\lambda_{k}\frac{r+m}{m}\prod_{p=1}^{C}(1-I(e_{p},e_{i},\pi_{k})E_{N_{p},A_{i,r,m}}))=\frac{r+m}{m}A_{i}$$

The output link and E2E PLR on the path $\pi_{k}$ is given as in Equation (5).

(5) $$B_{i,r,m}=E_{N_{i},A_{i,r,m}}=\frac{\left({\left(N_{i}A_{i,r,m}\right)}^{N_{i}}/N_{i}!\right)}{{\sum_{j=0}^{(N_{i}}N_{i}A_{i,r,m})}^{j}/j!}\;B(\pi_{k},r,m)=1-\prod_{e^{k}}(1-B_{i,r,m})$$

When a Bernoulli trial with probability of being lost $B(\pi_{k},r,m)$ as given in Equation (5), is assumed, the probability for *s* lost Data Packets and *r* lost Redundancy Packets is Binomial distributed, and given in Equation (6)) provided that the packet loss is independent:

(6) $$Q_{s}=\left(\begin{array}{c} m\\ s \end{array}\right)B{\left(\pi_{k},r,m\right)}^{s}(1-B{\left(\pi_{k},r,m\right)}^{m-s})R_{s}=\left(\begin{array}{c} r\\ s \end{array}\right)B{\left(\pi_{k},r,m\right)}^{s}(1-B{\left(\pi_{k},r,m\right)}^{r-s})$$

Equation (6) gives the probability for lost Data Packets (DP) and Redundancy Packets (RP) before a possible reconstruction has been done. However, as lost data packets might be recovered from successfully received DP and RP arrivals, the number of lost DPs may be decreased. It is also important to note that lost data packets after recovery lost DPAR ≤ lost DP. If the total number of lost DPs (*i*) and lost RPs (*j*) in a set is greater than the total number of transmitted RPs (*r*), recovery of data packets is not possible. Otherwise, reconstruction is possible, and there are no lost DPAR, as summarized in Algorithm 2. We set up the mean number of lost DPAR considering a packet set consisting of *m* DPs and *r* RPs transmitted on the path $\pi_{k}$ by using Equation (6):

(7) $$T_{m}(\pi_{k},r,m)=\sum_{i=1}^{m}\sum_{j=Max[r-i+1,0]}^{r}iQ_{i}R_{j}=\sum_{i=1}^{m}\sum_{j=Max[r-i+1,0]}^{r}i\left(\begin{array}{c} m\\ i \end{array}\right){\left(1-\prod_{e^{k}}(1-B_{i,r,m})\right)}_{i}\;\;\times {\left(\prod_{e^{k}}(1-B_{i,r,m})\right)}_{m-i}\left(\begin{array}{c} r\\ j \end{array}\right){\left(1-\prod_{e^{k}}(1-B_{i,r,m})\right)}_{j}{\left(\prod_{e^{k}}(1-B_{i,r,m})\right)}_{r-j}$$

**Algorithm 2:**
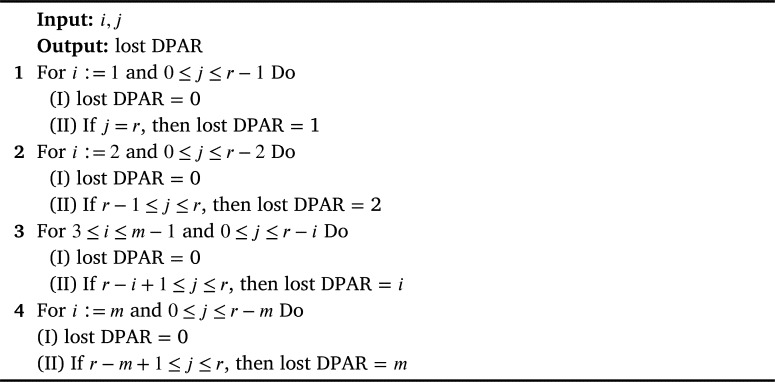
Lost DPAR as a function of Lost DPs (*i*) and RPs(*j*).

Considering the redundancy effect, the E2E PLR for path $\pi_{k}$ is then:

(8) $$T(\pi_{k},r,m)=\frac{1}{m}T_{m}(\pi_{k},r,m)$$

Equation (8) gives the DPLR on a single route in the optical network. The average PLR in the network is calculated by considering the PLR on all paths in the network. The RPLR and DPLR for the considered network are calculated as

(9) $$RPLR_{NET}=\frac{1}{\lambda_{TOT}}\sum_{\pi_{k}\in R}\lambda_{k}B(\pi_{k})DPLR_{NET}=\frac{1}{\lambda_{TOT}}\sum_{\pi_{k}\in R}\lambda_{k}T(\pi_{k},r,m)\;\text{where }\lambda_{TOT}=\sum_{\pi_{k}\in R}\lambda_{k}$$

### 3.5 CPT techniques

The scheme exploits non-systematic Reed Solomon code and path diversity to provide a non-cryptographic secrecy and low packet loss rate when contentions or path failures occur [24]. It presents the relationship between performance PLR, secrecy (ability to withstand targeted); and survivability (link failure). However, this relation introduces a considerable processing delay. The most important notations used to describe Reed Solomon code based CPT scheme are listed in Table 2.

**Table 2:** Definition of parameters.

Description	Symbol
Galois field	GF(2^*q*^)
Pkt loss threshold	*p*_*thres*_
Incoming node of the optical network	*n*_*i*_
Outgoing node of the optical network	*n*_*e*_
Uncoded pkts	*k*
Coded pkts	*n*
Redundant pkts	*r*
Pkts length	*L*
Disjoint routes	*l*
Uncoded pkts sent over disjoint routes	*m*
Coded packets sent over a disjoint routes	*m*′
PLR	*p*
Pkts overhead	*o*

**Figure 5:**
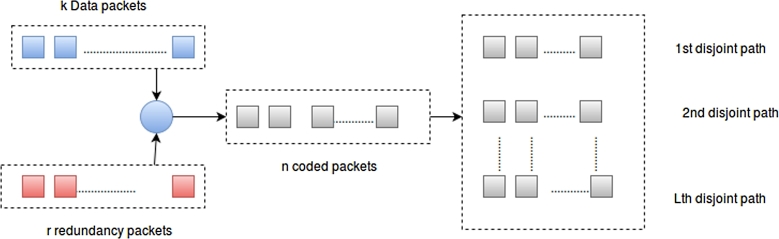
Illustration of CPT scheme illustration.

CPT scheme makes use of (n,k) Reed Solomon (RS) code as an erasure code and path diversity to recover packet loss due to contentions and path failure in optical networks [24]. To achieve a non-cryptographic secrecy, an optimal coding matrix computed over GF ($2^{q}$), where $n=2^{q}-1$ is used. The working principle of CPT scheme for improving the overall performance of optical networks is: Fig. 5, an OBS/OPS network which comprises the incoming and outgoing node, when original data packets arrive at the source node, it is encoded and assembled to form a coded packet. This means that the source node creates *n* encoded packets from a set of *k* data packets and *r* redundant packets. It then forwards the coded packet over multiple disjoint route to the destination node. The n×k encoding matrix for Reed Solomon code is given by:

$$G=\left[\begin{array}{cccccccc} 1 & \beta_{1}^{1} & \beta_{1}^{2} & \beta_{1}^{3} & . & . & . & \beta_{1}^{k-1}\\ 1 & \beta_{2}^{1} & \beta_{2}^{2} & \beta_{2}^{3} & . & . & . & \beta_{2}^{k-1}\\ 1 & \beta_{3}^{1} & \beta_{3}^{2} & \beta_{3}^{3} & . & . & . & \beta_{3}^{k-1}\\ . & . & . & . & . & . & . & .\\ . & . & . & . & . & . & . & .\\ . & . & . & . & . & . & . & .\\ 1 & \beta_{n}^{1} & \beta_{n}^{2} & \beta_{n}^{3} & . & . & . & \beta_{n}^{k-1} \end{array}\right]$$

The uncoded data packets are represented as a matrix where every row represents one data packet:

$$X=\left[\begin{array}{cccccccc} w_{1},{}_{1} & w_{1},{}_{2} & w_{1},{}_{3} & w_{1},{}_{4} & . & . & . & w_{1},{}_{L}\\ w_{2},{}_{1} & w_{2},{}_{2} & w_{2},{}_{3} & w_{2},{}_{4} & . & . & . & w_{2},{}_{L}\\ w_{3},{}_{1} & w_{3},{}_{2} & w_{3},{}_{3} & w_{3},{}_{4} & . & . & . & w_{3},{}_{L}\\ . & . & . & . & . & . & . & .\\ . & . & . & . & . & . & . & .\\ . & . & . & . & . & . & . & .\\ w_{k},{}_{1} & w_{k},{}_{2} & w_{k},{}_{3} & w_{k},{}_{4} & . & . & . & w_{k},{}_{L} \end{array}\right]$$

Finally, the coded packet at the ingress node is obtained as follows:

$$Y=\left[\begin{array}{cccccccc} w_{1},{}_{1} & w_{1},{}_{2} & w_{1},{}_{3} & w_{1},{}_{4} & . & . & . & w_{1},{}_{L}\\ w_{2},{}_{1} & w_{2},{}_{2} & w_{2},{}_{3} & w_{2},{}_{4} & . & . & . & w_{2},{}_{L}\\ . & . & . & . & . & . & . & .\\ . & . & . & . & . & . & . & .\\ {w_{m}}^{\prime },{}_{1} & {w_{m}}^{\prime },{}_{2} & {w_{m}}^{\prime },{}_{3} & {w_{m}}^{\prime },{}_{4} & . & . & . & {w_{m}}^{\prime },{}_{L}\\ w_{m^{\prime }+1},{}_{1} & w_{m^{\prime }+1},{}_{2} & w_{m^{\prime }+1},{}_{3} & w_{m^{\prime }+1},{}_{4} & . & . & . & w_{m^{\prime }+1},{}_{L}\\ w_{m^{\prime }+2},{}_{1} & w_{m^{\prime }+2},{}_{2} & w_{m^{\prime }+2},{}_{3} & w_{m^{\prime }+2},{}_{4} & . & . & . & w_{m^{\prime }+2},{}_{L}\\ . & . & . & . & . & . & . & .\\ . & . & . & . & . & . & . & .\\ w_{2m^{\prime }},{}_{1} & w_{2m^{\prime }},{}_{2} & w_{2m^{\prime }},{}_{3} & w_{2m^{\prime }},{}_{4} & . & . & . & w_{2m^{\prime }},{}_{L}\\ . & . & . & . & . & . & . & .\\ . & . & . & . & . & . & . & .\\ w_{(l-1)m^{\prime }+1},{}_{1} & w_{(l-1)m^{\prime }+1},{}_{2} & w_{(l-1)m^{\prime }+1},{}_{3} & w_{(l-1)m^{\prime }+1},{}_{4} & . & . & . & w_{(l-1)m^{\prime }+1},{}_{L}\\ . & . & . & . & . & . & . & .\\ . & . & . & . & . & . & . & .\\ w_{n},{}_{1} & w_{n},{}_{2} & w_{n},{}_{3} & w_{n},{}_{4} & . & . & . & w_{n},{}_{L} \end{array}\right]$$

## 4 Characteristic and performance data

### 4.1 Comparative analysis

#### 4.1.1 CPT

**Figure 6:**
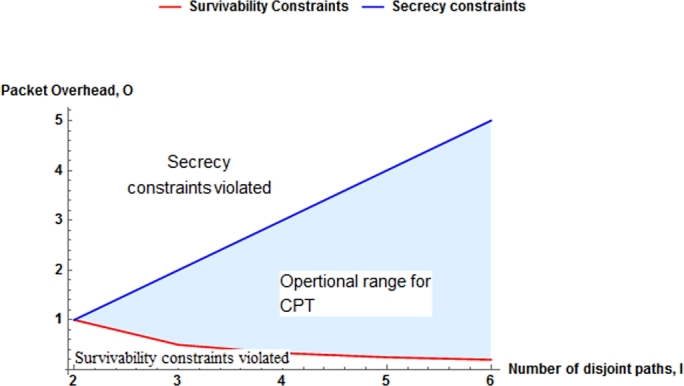
Plot of operational range of CPT.

With CPT scheme, the combined benefits of source coding by erasure codes and path diversity are employed to provide packet recovery due to contention and node failure and to provide non-cryptography secrecy. The overhead ratio of 1+1 and 1:N protection is 1 because the redundant and data packet's are same. When the CPT scheme is in its defined operational range shown in Fig. 6, it provides better performance compared to 1+1 or 1:N protection. The resulting operational rage conforms that the number of disjoint path is ≥ 3 or the packet overhead ratio is greater than 0.5. This is because the scheme offers both secrecy and survivability with lower packet overhead than 1+1 protection for single link failure.

Survivability and secrecy constraints are analyzed to define the operational range of the CPT, where survivability and secrecy are not violated as shown in Fig. 6. For example, let us consider the number of disjoint paths l=3, packet overhead ratio o=1 and RS code (24,12) over GF(2^8^) which implies that k=12 and r=12. To achieve both survivability and secrecy, 8 packets are sent over the three disjoint paths. In this case, an external adversary gets only 8 packets from a single node/link compromise. To recover the whole data packet, he needs 4 more packets. Since the coding is performed over GF(2^8^), he needs brute force search of $2^{4⁎8}=2^{32}$ tries which is a bit exhaustive.

#### 4.1.2 NPLRS

To illustrate NPLRS numerically, suppose $m_{s}=8$ and $r_{s}=2$, $m_{r}=6$, $r_{r}=2$. In this example reconstruction is possible since $m_{r}+r_{r}=6+2=8\geq m_{s}=8$ which results in zero lost packets when NPLRS. This implies that the scheme depends on the values of $m_{s}$ and $r_{s}$, the offered load, numbers nodes traversed, data packet arrived process and packet length distribution. When the above parameter setting (or offered system load) on the network is increased further, then the scheme increases burstiness. If utilized in a suited setting, NPLRS, the potential benefit of reducing the plr for data packets.

Considering the m/r constant, rising the value of m enhances the system performance. But this happens at the cost of more end-to-end delay because the packet size increases. More network load also degrades the performance as the packet arrival processing affects the network performance. However, it is important to note that NPLRS is not a contention resolution scheme and can be combined with wavelength conversion mechanism to resolve contentions.

#### 4.1.3 SPLR, HPLR, and Error concealment

**Figure 7:**
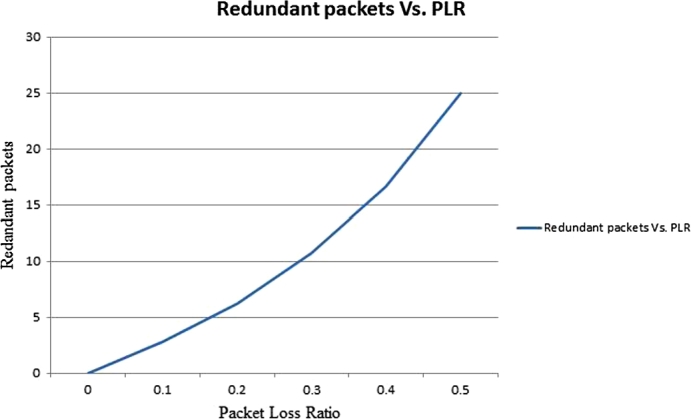
Plot of redundant packets versus PLR.

According to the author [13], the SPLR scheme combines the two basic QoS improving scheme, Error concealment and HPLR at the same time to recover all packet lost during network transmission. This technique has a low probability of packet loss as it allows sharing of redundant packet at the ingress node for packet recovery. To illustrate, consider an ingress node having a bandwidth (the number of packets that is coming out of ingress node in this case) of n=30 per packetization period, it applies an erasure code on k=25, and adds r=5 more packets. Thus, the ingress node forwards these 30 packets over a network to the egress node. The egress node has to receive any of 25 packets from the 30 packets for recovering the lost packets from the data packets. We provide analytical model for the average number of redundant packets provided that the PLR is *p* and the loss of the redundant packets is accounted. The relationship between *p* and *r* is illustrated in Fig. 7.

(10) $$r=\frac{kp}{1-p}$$

where *r* = the average number of redundant packets, *k* = data packets and *p* = packet loss ratio.

We noticed that, in CPT scheme, the mechanism used to overcome packet loss due to contention is burst segmentation that means a single file is fragmented into the number of disjoint paths. The increasing number of Internet services especially video streaming broadcasting), e-banking, 3D-TV, e-voting and emergency services requires a high demand on QoS of an OBS/OPS network. Hence, we analyzed the QoS requirements of CPT scheme for the future optical core network. Currently, telecommunication systems are using various redundancy schemes to improve their performance. Considering the discussions, the authors believe that the NLPRS and CPT to be a feasible approach to enhance performance in OPS networks.

### 4.2 Characteristic analysis

Table 3 presents the comparison of CPT scheme and other state of art methods both for IP networks and optical networks. The comparison depends on which scheme provides low Packet Loss Rate (PLR), secrecy and survivability. The techniques that can be employed in each method are also included.

**Table 3:** Comparison of CPT scheme and other methods, where * indicates yes in case of database system.

Parameters	Error concealment	SPLR	HPLR	NLPRS	CPT
Erasure coding	Not required	Applied on all streams	Depends on the correlation of preceding or succeeding packets	Required	Required

Redundant packets	Not required	Shared among stream	Not required	Required	Required

Network	IP networks	IP networks	IP networks	OPS network	OPS/OBS network

PLR	Yes	Yes	Yes	Yes	Yes

Secrecy	No	No	No	No	Yes

Survivability	No	No	No	*yes*^⁎^	Yes

It is observed that Error concealment recovers the lost packets in a networking by correlating the preceding and/or succeeding packets of the same data set and can only reduce the probability of packet loss. However, SPLR scheme does the function by using an erasure code to add redundant packets in the ingress node of a network to reduce the lost packets. The third technique of recovering the lost packets in a network is HPLR which combines the important features of both Error concealment and SPLR. This means that the scheme uses error concealment to recover lost packets to reduce the unnecessary sending of redundant packets. If the correlation between preceding and/or succeeding packets is not sufficient, HPLR applies SPLR. Advancements such as NLPRS and CPT scheme try to reduce the PLR and other bottlenecks in networks. NPLRS scheme provides survivability and recover packets lost due to contentions and node/link failures in OPS/OBS networks. Besides, CPT scheme also recovers the lost packet due to contention and node failures while providing secrecy and survivability in optical networks. The two techniques come up with interesting result with regard to both PLR and survivability at the expense of an increased processing delay. Compared to Error concealment, SPLR and HPLR, both NLPRS and CPT link the interaction between PLR and survivability.

As per NIST requirement in 2015, for robust security, the level of security should be between 80 and 112 bits. Thus, the paper uses these modern recommended levels of security for evaluating CPT scheme with regard to secrecy.

As a general conclusion for CPT scheme, we observed that certain amount of packet overhead needs to be provided for ensuring protection against node/link failure and survivability. Furthermore, for guaranteeing secrecy against eavesdropping, amount of data packets to be send should not be less than *k* on each path.

## 5 Conclusion

This work presented the comparison of CPT and other methods used for improving the performance of optical networks. The background study of different methods used to meet QoS requirement in OBS and/or OPS networks were also explained. Furthermore, the different states of art methods proposed by other authors are compared with CPT scheme. A feasibility study of QoS in optical network has also been conducted. The concept and implementation of CPT scheme is also explained.

## 6 Glossary of terms

1+1**protection**: a protection mechanism where the signal is simultaneously transmitted over two paths.
1:1**protection**: a protection mechanism where the signal is transmitted over a working path under normal conditions but switched to a protect path after a failure.
1:N**protection**: a more compact form of 1:1 protection in which *N* working paths share a protection path.
**Burst**: refers to a set of packets with similar properties. The assembly of IP packets into bursts and dissembling occurs at the edge routers.
**Disjoint paths**: refers to node disjoint paths between two nodes in a network.
**FEC**: Forward Error Correction is a technique which adds redundant information to the original message, so that corrupted data can be recovered at the destination, using the added redundant information.
**Galois field**: also known as finite field consisting of finite elements that are generated from a primitive element, denoted by *α*.
**Offset time**: is the time interval between the BCH and Date Burst (DB) for maintaining the required resources.
**Service level agreement**: the agreement made between customers and operators/service provide on service specification.
**Survivability**: the ability of a network to provide service in the presence of failures, link and node failure.
**User**: someone who has access to the internet at home.

## Declarations

### Author contribution statement

Dawit Hadush Hailu: Conceived and designed the experiments; Wrote the paper.

Gebrehiwet Gebrekrstos Lema: Performed the experiments.

Berihu G. Gebrehaweria: Analyzed and interpreted the data.

Samrawit H. Kebede: Contributed reagents, materials, analysis tools or data.

### Funding statement

This research did not receive any specific grant from funding agencies in the public, commercial, or not-for-profit sectors.

### Competing interest statement

The authors declare no conflict of interest.

### Additional information

No additional information is available for this paper.
